# Three‐Dimensional Printing in Breast Radiation Therapy: A Scoping Review of the Literature

**DOI:** 10.1002/jmrs.70000

**Published:** 2025-06-27

**Authors:** Chamitha Weerasinghe, Patrick Estoesta, Ashley Cullen, Elizabeth Claridge Mackonis

**Affiliations:** ^1^ Department of Radiation Oncology Chris O'Brien Lifehouse Camperdown New South Wales Australia; ^2^ Royal Prince Alfred Hospital Camperdown New South Wales Australia; ^3^ Radiation Oncology, Crown Princess Mary Cancer Centre Westmead Hospital Sydney New South Wales Australia; ^4^ Radiation Oncology, Blacktown Cancer and Haematology Centre Blacktown Hospital Blacktown New South Wales Australia; ^5^ Affiliate, Sydney Medical Program (Westmead Clinical School), Faculty of Medicine and Health The University of Sydney Sydney New South Wales Australia; ^6^ Institute of Medical Physics, School of Physics University of Sydney Camperdown New South Wales Australia

**Keywords:** 3D printed bolus, 3D printing, breast, chest wall, radiation therapy, radiotherapy, three‐dimensional printing

## Abstract

**Introduction:**

Tissue equivalent bolus is used to increase dose to skin and superficial tissue in adjuvant breast or chest wall radiation therapy. Three‐dimensional (3D) printed bolus offers a customised conformal device and potential for improved anatomic conformity and dose distribution.

**Methods:**

A literature search was defined as per PRISMA guidelines for scoping review. Inclusion criteria were determined using PICO guidelines. The parameters of interest, including year, journal, study type, intervention details, clinical sample size and pre‐clinical case numbers, were extracted from each article. The reported outcomes, such as dosimetry, anatomic conformity, dose to organs at risk and toxicity data were recorded.

**Results:**

Thirteen publications were reviewed, six studies were pre‐clinical and seven were clinical. Ten were performed in the post‐mastectomy setting and utilised polylactic acid (PLA) bolus. Overall, most studies reported marginal improvement in dosimetry, anatomic conformity, organ at risk dosimetry and toxicity with 3D printed bolus. However, sample sizes utilised were small and study design was variable with unusual choices of comparator arm and introduction of other variables.

**Conclusion:**

Three‐dimensional printed bolus is an emerging technology in radiation oncology. Most available data in the setting of breast radiation therapy is positive, though interpretation of results is difficult given the small sample sizes and variable study design. Further investigations in larger cohorts in a clinical setting is warranted.

## Graphical abstract

Three‐dimensional (3D) printed bolus is an emerging customisable device with potential for improved conformity and dose distribution in comparison to the commercially available alternatives. This scoping review performs an analysis of the pre‐clinical and clinical evaluation of 3D printed bolus in breast radiation therapy.

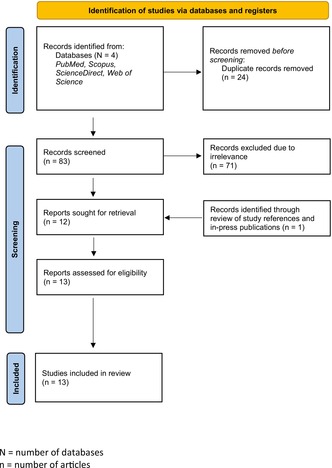

## 1 Introduction

Radiation therapy plays a crucial role in the treatment of breast cancer to reduce the risk of local recurrence and improve overall survival [1]. Bolus is utilised in radiation therapy to modify the dose distribution and achieve adequate dose to the skin surface and superficial tissues.

Historically, traditional bolus materials such as wax, standard vinyl gel sheet (Superflab), and wet gauze have been widely employed. The potential limitations of these materials, such as lack of reproducibility, difficulty in conforming to the patient's contour, and potential for air gaps [2] have prompted the exploration of innovative solutions to enhance the precision and effectiveness of bolus application. With the rapid advancements in additive manufacturing technologies, three‐dimensional (3D) printed bolus has emerged as a promising alternative. This technique enables the construction of customised bolus devices, offering potential for improved dose conformity.

The use of bolus in breast radiation therapy is particularly relevant in three distinct clinical contexts: post‐mastectomy chest wall irradiation with and without tissue expanders and post‐breast‐conserving surgery. These settings have necessitated a tailored approach to bolus application. Radiation techniques used include megavoltage photons in tangential fields, intensity modulated radiation therapy (IMRT) and volumetric modulated radiation therapy (VMAT), with or without either a photon or electron boost.

The aim of this investigation is to evaluate the current assessment and application of 3D printed bolus in breast radiation therapy by conducting a scoping review of the literature as per the Preferred Reporting Items for Systematic Reviews and Meta‐Analyses (PRISMA) [3] guidelines. The objectives are to review the pre‐clinical and clinical evaluation of 3D printed bolus and characterise current clinical use in adjuvant breast or post‐mastectomy radiation.

## 2 Methods

### 2.1 Literature Search

The literature search for this scoping review was conducted following the PRISMA [3] guidelines. A comprehensive search strategy was devised to identify relevant articles. One independent reviewer (CW) performed the search across four electronic databases: PubMed, Scopus, ScienceDirect and Web of Science. The pre‐determined search terms utilised a combination of Boolean terminology and keywords, including (‘radiation therapy’ OR ‘radiotherapy’) AND ‘bolus’ AND (‘3D print’ OR ‘three‐dimensional print’) AND breast. References of included articles were reviewed to identify additional articles for inclusion.

### 2.2 Inclusion and Exclusion Criteria

The inclusion criteria for article selection were based on the population, intervention, control and outcomes guidelines [4]. The population included peer‐reviewed journal articles, reviews and guidelines available in the English language from January 1, 2007 to May 30, 2023, to capture the period of 3D printing technology investigation or use in radiation therapy. Publications that compared the use of 3D printed bolus to either traditional bolus (standard vinyl gel sheet), treatment planning software (TPS) calculated bolus or historical data in the post‐mastectomy or post‐breast‐conserving surgery radiation therapy setting were included. The outcomes of interest encompassed qualitative and quantitative data, including dosimetry, anatomic conformity, dose to organs at risk (OARs), toxicities and workflow processes. The initial search was conducted from April 2023 to May 2023. The retrieved articles were screened based on their titles and abstracts to identify potentially relevant studies. Full‐text articles were obtained for further assessment.

### 2.3 Data Extraction and Analysis

Title and abstract review was performed by an independent reviewer (CW) and the rationale for exclusion of articles was reviewed by all authors. A full text review of the included articles was performed by all authors, and data endpoints were entered into a structured Excel database. Authors consist of a multidisciplinary team including physicists, radiation therapists and radiation oncologists. Parameters of interest, including year, journal, study type, intervention details (bolus type, radiation type and technique), clinical sample size and pre‐clinical case numbers, were extracted from each article. The reported outcomes, such as dosimetry, anatomic conformity, dose to OAR and toxicity data, were recorded.

## 3 Results

**FIGURE 1:**
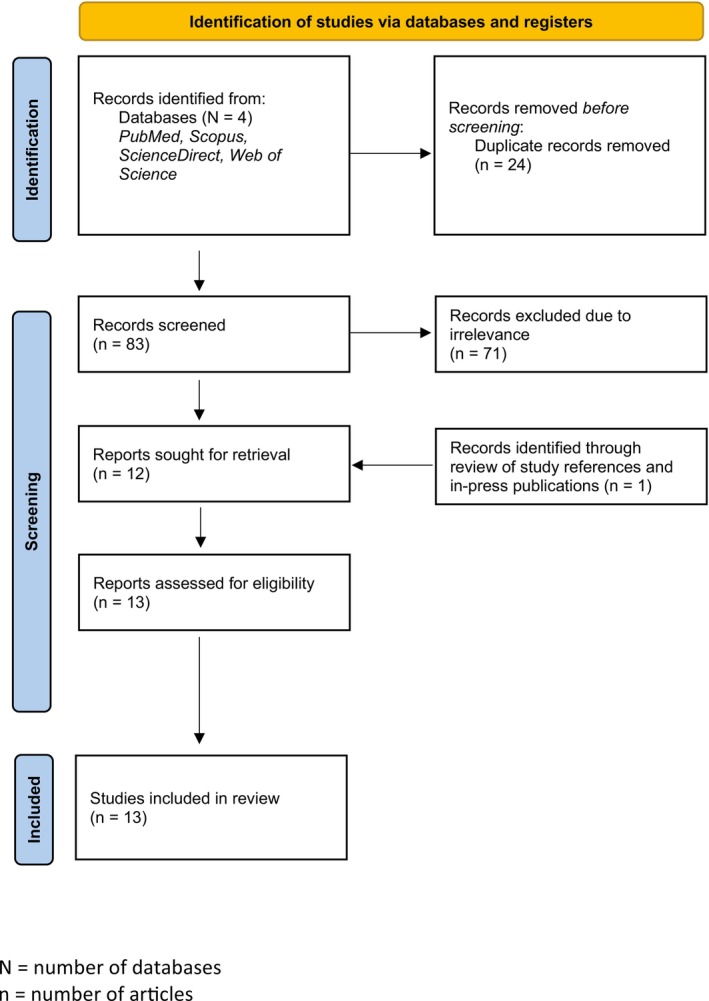
Literature search protocol for identification of articles as per PRISMA guidelines.

Literature searches resulted in 107 articles across the four databases; 83 articles were available for review after the removal of duplicates. Twelve articles met the inclusion criteria, and one additional article was identified through the review of study references and in‐press publications. In total, 13 articles were included for scoping review (Figure 1).

Seven of the identified articles for review were clinical studies; the sample size for these articles was generally small, ranging from 6 to 75, with one outlier of 360 patients; though in this study, only a subset of 27 patients underwent dosimetric comparison. Five articles utilised megavoltage photon radiation therapy with IMRT or VMAT. Ten of the articles were in the post‐mastectomy setting, two in the adjuvant wide local excision/breast conserving surgery (BCS) setting and one in the intra‐operative setting. Eleven of the articles delivered conventional or moderately hypofractionated radiation therapy. Among the 13 reviewed articles, PLA bolus was included in 10 studies, whereas acrylonitrile butadiene styrene, thermoplastic elastomer (TPE), silicone Hydrogel and silica/epoxy bolus materials were only included in one study. Table 1 outlines an evaluation summary of the identified articles. A detailed description of individual studies is available in Table 2 [5, 6, 7, 8, 9, 10, 11, 12, 13, 14, 15, 16, 17].

**TABLE 1:** Evaluation summary of identified articles.

Descriptor	Number of studies
**Study type**	
Pre‐clinical	6
Clinical	7
**Radiation setting**	
Post‐mastectomy	10
Breast conserving surgery	2
Intra‐operative electron radiation therapy	1
**Radiation type and technique**	
Photon IMRT/VMAT	11
Electron	3
Electron IOERT	1
**Bolus type**	
PLA	10
TPE	1
ABS	1
Silicone hydrogel	1
Silica/epoxy	1
**Outcome**	
Dosimetry	12
Anatomic conformity	5
OAR	8
Toxicity	4
Workflow/process	2
Adhesion	1

Abbreviations: ABS, acrylonitrile butadiene styrene; IMRT, intensity modulated radiation therapy; OAR, organ at risk; PLA, polylactic acid; TPE, thermoplastic elastomer; VMAT, volumetric modulated radiation therapy.

**TABLE 2:** Summary of studies evaluating 3D printed bolus in the breast radiotherapy setting.

Year	Author	Study type	Intervention/application	Bolus Type	Radiotherapy technique	Number of patients	Outcomes
2016	Ha J [5]	Pre‐clinical	Standard vinyl gel sheet and PLA comparison on anthropomorphic phantom	PLA	PMRT (post reconstruction) Photon; Tomo‐IMRT or Tomo‐Direct		Air gaps: Conventional: 0.8 cm minimum, 1.5 cm maximum 3DPB: Unable to assess (well contacted with phantom surface, water equivalent gel used to eliminate air gap for 3DPB) Mean dose: Conventional (cGy): measured 174.5, TPS calculated 173.5 3DPB (cGy): measured 178.7, TPS calculated 177 Lung *D*_Mean_ Tomo‐IMRT (Gy):10.02 ± 7.59 conventional, 8.68 ± 7.31 3DPB Tomo‐direct (Gy): 2.98 ± 7.56 conventional, 2.66 ± 6.71 3DPB
2023	Lee V [6]	Pre‐clinical	Hypofractionated photon RT with electron boost with and without 3DPB on retrospective patient dataset	PLA	BCS photon; tangent electron	27	CI: 0.48 ± 0.9 with no bolus, 0.56 ± 0.72 (3DPB) *D*_max_ LAD: 19.04 ± 8.48 with no bolus, 17.75 ± 7.60 3DPB Ipsilateral lung: V_5Gy_ 23.48% ± 7.83% with no bolus, 19.34% ± 6.37% 3DPB V_20Gy_ 9.06% ± 3.72% with no bolus, 8.31% ± 3.62% 3DPB
2018	Dipasquale G [7]	Pre‐clinical	3D surface scanner and CT assisted 3DPB production comparison on phantom and volunteers	PLA ABS Resin	BCS Photon	NA	30 profiles (anthropomorphic phantom and clinical) Air gaps (phantom): CT assisted 1–2 mm, surface scanner: < 0.6 mm Qualitative assessment—improved smoothness and fit Various anatomical sites, no separate breast data included
2021	Zhang C [8]	Pre‐clinical	Silicone hydrogel bolus and CT calculated bolus comparison on anthropomorphic phantom	Silicone hydrogel	PMRT Photon; IMRT	NA	Absolute dose difference < 0.12% (minimum 0.02%, maximum 1.17%) Adhesion to phantom: solid water phantom: 1.3% three anthropomorphic phantoms: 7.0%, 3.0% and 3.4% Mean air gap volume 2.9 cc, maximum air cavity dimension 2.6 mm OAR dosimetric difference < 2.4%
2020	Moghaddam S [9]	Pre‐clinical	3DPB and water phantom comparison	PLA	Electron; IOERT	NA	Mean PDD for PLA versus water phantom 0.9%, 0.4%, 0.8%, 0.2% for 6, 8, 10 and 12 MeV, respectively Mean gamma index 0.3, 0.2, 0.5 and 0.1, respectively
2016	Park S [10]	Pre‐clinical	Vinyl gel sheet and 3DPB comparison on anthropomorphic phantom	PLA	PMRT Photon; tangent with electron boost	NA	Percentage dose difference between calculated and measured dose −0.5% to −0.9% 3 mm PLA, −5.1% (−3.8% to −7.6%) 3 mm vinyl sheet −1.1% 5 mm PLA, −3.2% (−2.7% to −3.5%) 5 mm vinyl sheet PLA bolus described as ‘good fit’ with no air gaps, unable to use PLA for larger breast volume (> 400 cc) due to poor fit at inframammary fold
2022	Gong P [11]	Clinical	Single arm TPE and historical conventional data comparison (Zhang et al. [12])	TPE	PMRT Photon; IMRT/VMAT	32	D98% 4879.13 cGy, D95% 4997.06 cGy, D2% 5381.75 cGy CI of PTV 0.75, HI of PTV 0.10 Heart *D*_mean_ 348.69 cGy RT dermatitis (30 patients reviewed): G1: 3 (10%) Week 4, 14 (46.7%) Week 5, 70% Week 7. G2: 13.3% Week 7 Depression: 2 patients in Week 3 and Week 5 Anxiety: 1 in Weeks 1, 3 and 5
2023	Zhang Y [13]	Clinical	Standard vinyl gel sheet and 3DPB comparison	PLA	PMRT Photon; VMAT	75	PTV V_50Gy_: 95.8% ± 0.6% conventional, 94.8% ± 0.8% 3DPB, *p* = 0.026 PTV CI: 0.83 ± 0.02 conventional, 0.80 ± 0.03 3DPB Heart *D*_mean_ (Gy): 5.5 ± 1.3 conventional, 4.7 ± 0.8 3DPB, *p* = 0.035 Ipsilateral lung *D*_mean_ (Gy)12.4 ± 1.0 conventional, 11.6 ± 0.8 3DPB, *p* < 0.001 > G2 RT dermatitis:17/40 conventional, 12/35 3DPB, not statistically significant G1 RT pneumonitis: 20% conventional, 14.3% 3DPB >G2 RT pneumonitis: 3/40 conventional, 0 3DPB
2022	Wang X [14]	Clinical	Pre‐clinical evaluation, clinical evaluation of 3DPB	PLA	PMRT Photon; IMRT	360 27 for dosimetric evaluation	CI 99.94% (97.41%–100%), HI 0.07 (0.06–0.17) Median surface dose: calculated 208.85 cGy (203.16–212.53 cGy), measured 209.53 cGy (204.14–214.42 cGy) Ipsilateral lung: *D*_mean_ 1341 cGy (1208–1385 cGy), V_20_ 24.55% (21.58%–26.93%), V_5_ 48.06% (39.75%–48.97%) Heart: *D*_mean_ 339 cGy (138–640 cGy) Radiation dermatitis: G2–4 41.67% (64.7% G2)
2019	Yang K [15]	Clinical	Electron therapy with 3DPB and conventional photon tangent comparison	PLA	PMRT Electron Photon; tangents	28	Treated with electron RT, dosimetric comparison to photon RT Heart; *D*_mean_ (Gy): 4.33 conventional, 1.94 3DPB; *p* < 0.001 *D*_max_ (Gy): 49.41 conventional, 34.24 3DPB; *p* < 0.019 ipsilateral lung; *D*_mean_ (Gy) 13.46 conventional, 8 3DPB V_20Gy_ 28.45% conventional, 16.82% 3DPB V_5Gy_ 42.29% conventional, 32.63% 3DPB Toxicity: G1 RT dermatitis 1 patient, lymphoedema 4 patient, fibrotic lung changes 1 patient
2017	Park K [12]	Clinical	Vinyl gel sheet and 3DPB comparison	PLA	PMRT Electron	6	CI: 0.72 conventional, 0.81 3DPB; HI: 2.61 conventional, 2.51 3DPB Lung *D*_mean_ (Gy): 9.15 conventional, 8.24 3DPB; Lung *D*_median_ (Gy): 9.06 conventional, 7.99 3DPB Heart *D*_mean_ (Gy): 4.13 conventional, 3.80 3DPB Heart *D*_median_ (Gy): 4.82 conventional, 4.48 3DPB
2018	Robar J [16]	Clinical	Standard vinyl gel sheet and 3DPB comparison	PLA	PMRT Photon; hybrid IMRT	16	Mean coefficients of dose variation: 0.028 conventional, 0.029 3DPB Mean air gap: sheet bolus 0.5 ± 0.3 cm, 3DPB 0.3 ± 0.3 cm One outlier 2.2 cm (patient contour change over duration of RT) Percentage of air gaps > 5 mm: 30% conventional, 13% 3DPB Clinical set up (seconds): 104 conventional, 76 3DPB *Note:* $10 per print, median and mean print time: 10.8 and 12.6 ± 5.4 h respectively, range 7.2–28 h 4 of 16 boli required > 15 h
2020	He C [17]	Clinical	No bolus, conventional bolus and 3D precise breast conformer (3DPB and fixing device) comparison	Silica/epoxy	BCS Photon; IMRT	30	3D surface scanner (mould generated, silica/epoxy injected) Increased V_95%_ and V_98%_ with conventional bolus and 3DPB 3DPB: HI 0.08 ± 0.03, CI 0.95 ± 0.03 Conventional: HI 0.15 ± 0.05, CI 0.87 ± 0.04 No bolus: HI 0.34 ± 0.07, CI 0.78 ± 0.06

Abbreviations: 3DPB, 3D printed bolus; BCS, Breast conserving surgery; CI, Conformity Index; CT, computed tomography; G1, Grade 1; G2, Grade 2; HI, Homogeneity Index; IMRT, intensity modulated radiation therapy; LAD, left anterior descending artery; PMRT, post‐mastectomy radiation therapy; RT, radiation therapy; VMAT, volumetric modulated radiation therapy.

### 3.1 Adhesion of Bolus

Adhesion was demonstrated as 1.3% for a solid water phantom and 7.0%, 3.0% and 3.4% [8] for three separate anthropomorphic phantoms in a single study assessing Hydrogel silicone bolus.

### 3.2 Workflow and Production Cost

Print time for bolus production was reported in two studies, an average of 10^6^ h and a median of 10.8^16^ h was reported. The range of print times was 7.2–28 h per bolus. Clinical set up time was only reported in a single study, however reported a reduction from 104 to 76 s. Bolus production cost ranged from $26 to $1016 per bolus.

### 3.3 Anatomic Conformity

Pre‐clinical studies demonstrated a reduction in air gaps between the bolus and phantom. A qualitative assessment of a ‘good fit’ was provided with the use of PLA bolus with an anthropomorphic phantom [10], and when compared to conventional bolus, PLA was shown to have no air gap compared to air gaps of 0.8–1.5 cm. Silicon Hydrogel bolus showed an air gap volume of 2.9cc [8]. The presence of air gaps with surface scanner associated bolus production compared to CT calculated bolus production showed air gaps of < 0.6 mm and 1.0–2.0 mm respectively [7].

PLA bolus in the clinical setting demonstrated a mean air gap of 0.3 ± 0.3 cm compared to 0.5 ± 0.3 cm when compared to vinyl gel sheet in 16 patients [16].

### 3.4 Dosimetry

Surface dosimetry showed marginal increases in surface dose pre‐clinically. 3D printed PLA bolus and conventional bolus showed a surface dose of 178.7 cGy and 174.5cGy [5]. The percentage dose difference for calculated and measured surface dose for 3 mm bolus was 0.5%–0.9% for PLA compared to 5.1% for conventional bolus [10]. For the 5 mm bolus, the percentage dose difference was 1.1% and 3.2% for the PLA and conventional bolus respectively [10]. Silicone hydrogel also performed well pre‐clinically with an absolute dose difference of < 0.12% (range 0.02%–1.17%) [8].

Clinically small increases in dose delivered to the planning target volume (PTV) were seen with PLA compared to conventional bolus, 94.8% ± 0.8% compared to 95.8% ± 0.6% respectively, *p* = 0.026 [13]. A higher conformity index was also noted, 0.83 ± 0.02 compared to 0.80 ± 0.03, *p* < 0.001, for PLA and conventional bolus respectively. In vivo dosimetry showed a mean coefficient variation of 0.029 for PLA bolus and 0.028 for conventional bolus [16]. A dosimetric evaluation of 27 patients demonstrated a conformity index of 99.94% for PLA and a homogeneity index of 0.07 [14].

A 32 patient study of TPE bolus showed a conformity index of 0.75, and homogeneity index of 0.10 compared to historical data of 0.83 and 0.10, respectively [11]. Silica and epoxy injected mould 3D printed bolus showed improved homogeneity index and conformity indexes compared to conventional bolus, 0.08 compared to 0.15 for homogeneity and 0.95 compared to 0.87 for conformity for conventional and mould bolus, respectively [17].

PLA and conventional bolus in the setting of electron therapy showed a conformity index of 0.80 and 0.71, respectively and marginally higher mean homogeneity index of 2.61 and 2.51, respectively.

### 3.5 Dose Reduction to OAR

#### 3.5.1 Pre‐Clinical

Dose reduction to left anterior descending (LAD) artery by 3% and ipsilateral V5 and V20 by 4% and 2% respectively was seen with PLA in the setting of an electron boost in comparison to an electron boost with no bolus applied [6].

A comparison of conventional bolus to PLA demonstrated a reduction in mean ipsilateral lung dose with readings of 10.02 ± 7.59 Gy and 8.68 ± 7.31 Gy respectively for Tomo‐IMRT and 2.98 ± 7.56 Gy and 2.66 ± 6.71 Gy respectively for Tomo‐Direct radiation therapy [5].

A silicone Hydrogel bolus comparison to virtual CT bolus demonstrated a < 2.4% dosimetric difference to OAR [8].

#### 3.5.2 Clinical

A study of PLA bolus in 28 patients showed mean and maximum heart doses were 4.33 Gy and 49.41 Gy for conventional photon therapy and 1.94 Gy and 34.24 Gy for electron therapy, with *p*‐values < 0.001 and < 0.019 respectively. Mean, V_5_ and V_20_ ipsilateral lung doses were also reduced with the 3D printed bolus and electron therapy arm.

Another clinical study of PLA showed an on average reduction of 0.8 Gy to mean heart dose (*p* = 0.035) and ipsilateral lung dose (*p* < 0.001) with the use of PLA compared to standard vinyl gel sheet [13].

### 3.6 Toxicities

Seventeen of 40 patients with conventional bolus and 12 of 35 patients with 3D printed PLA bolus were found to have Grade 2 radiation dermatitis in the post‐mastectomy setting, this difference in toxicity rate was not statistically significant [13]. Grade 2–4 radiation dermatitis rates was 41.67% with PLA bolus in a separate study, 64.7% of which were Grade 2 [14]. Electron therapy with PLA bolus in a study of 28 patients showed radiation dermatitis in one patient, lymphoedema requiring intervention in four patients and fibrotic lung changes in one patient [15].

A single study of 30 patients demonstrated radiation dermatitis with TPE bolus. Three experienced Grade 1 radiation dermatitis in Week 4, 14 in Week 5 and 21 in Week 7, while four patients experienced Grade 2 radiation dermatitis in Week 7 [11].

Grade 1 radiation pneumonitis was reported as 20% compared to 14.3% in conventional and PLA bolus, and Grade 2 radiation pneumonitis was seen in three patients in the conventional bolus group and no instances in the PLA bolus group.

## 4 Discussion

To the best of our knowledge, this scoping review represents the only scoping review of 3D printed bolus application in the breast and post‐mastectomy radiation therapy setting. The use of 3D printed bolus in breast radiation therapy is of particular interest given the potential for high patient‐specific customisation, improved anatomic conformity, improved dose distribution and accuracy for skin and superficial tissues, OAR constraint optimisation and efficient and economical workflow. Anatomic contour changes due to post‐operative complications, such as oedema and seroma require consideration when applying bolus. The theoretical ability of 3D printed bolus to precisely match the unique anatomy of each patient is vital for this process, particularly in this setting of irregular breast and chest wall contour post‐operatively. Anthropomorphic phantoms provide an approximate simulation of variation in patient anatomy; therefore, more clinical investigations would be ideal to demonstrate clinical application. The improved conformity and subsequent reduction in air gaps contribute to improved dose distribution and target coverage during radiation therapy. Improved surface and superficial tissue dose are clinically relevant in cases at high risk of local recurrence, such as in the setting of positive margins or inflammatory breast cancer. The customisability of 3D printed bolus may then improve dose distribution, though this has not been established in current data [18].

Although the material flexibility and durability of 3D printed bolus have not been studied in the breast and post‐mastectomy setting, these features have been extensively explored for other anatomical sites, and they hold promise for additional benefits [19]. The ability to select materials, particularly in the indirect 3D printing process, may enhance treatment through variation in density of materials, adhesion to patient and flexibility.

### 4.1 Bolus Production: Construction, Workflow Processes and Cost

3D bolus is produced with either direct or indirect printing [20]. Direct printing involves obtaining the patient's contour via computed tomography (CT) or 3D surface scanner and using TPS to calculate bolus shape based on desired dose distribution [18]. This information is exported and converted into a digital format, commonly a Standard Tessellation Language (STL) file. A 3D printer produces a customised bolus using suitable materials as per the specifications of the STL [21]. Indirect printing involves using either CT or 3D surface scanner to capture the patient contour, production of a digital file and printing a customised mould. Materials, such as silica, epoxy or paraffin, are injected into the mould. This provides flexibility in material selection and generates a replica of the desired bolus shape.

Total print time for bolus production was recorded in two of the included studies, of which there was notable variation. Average and median print times of 10^6^ and 10.8^16^ h, respectively were reported; however, the range of print times extended from 7.2 to up to 28 h [16]. Four of 16 boluses required 15 h or longer. All boluses were printed using the same 3D printed equipment and processes; however, size and shape varied to accommodate patient anatomy. These print times are important to consider in the context of high‐volume production of boluses, though staff involvement would be significantly less than the print time, as the supervision of the printing process is minimal [16]. The total cost for 3D printed bolus was only reported in two publications. The range in bolus cost may be reflected by the infill percentage and subsequent density; the more expensive $10 bolus utilised a 100% infill with electron density of 3.8 × 10^23^ electrons/cm^3^ [16], while the less expensive $2 bolus did not specify an infill percentage but reported a physical density of 1.19 g/cm^3^ [6]. Unfortunately, the impact of variation in the size of the bolus was not discussed in either paper. The costs associated with the outlay of machinery, quality assurance and staffing were not reported. The improvement in clinical setup time of the bolus has not been widely investigated, though Robar et al. [16] demonstrated a reduction from 104 to 76 s.

### 4.2 Adhesion

Adhesion of bolus has only been assessed in a single study, Zhang et al. [8], which calculated adhesion as a percentage value of air gaps to bolus volume with the use of Hydrogel silicone bolus, a flexible 3D printed bolus material. The use of this calculation has not been validated. However, by this metric reasonable adhesion was demonstrated with 1.3% for a solid water phantom and 7.0%, 3.0% and 3.4% for three separate anthropomorphic phantoms. The interpretation of this is limited given the absence of comparison to a bolus, though this may be an area of further study should adhesive boluses be considered for 3D printed bolus production.

### 4.3 Anatomic Conformity

Poor anatomic conformity and subsequent air gaps can reduce radiation dose to the skin surface and superficial tissues [22]. Personalised 3D printed bolus has the potential for close conformity to the patient's skin and enhanced accuracy of radiation therapy dose delivery [23]. Pre‐clinical evidence demonstrated a reduction in air gaps with the use of PLA compared to conventional bolus. The rigid PLA bolus was described as having a ‘good fit’ [10], however, it should be noted that this study printed PLA bolus for a variety of anthropomorphic breast volumes, and the largest breast phantom used, > 400 cc, had a poor fit at the inframammary fold and was subsequently not included in the study. Unfortunately, the cause for the poor fit was poorly described. The application of rigid PLA bolus for larger breasted patients is unclear; however, these findings are concerning for potential poor fit and dosimetry, particularly in the setting of breast ptosis. Quantitatively, a complete absence of air gaps with 3D printed PLA bolus compared to air gaps of 0.8–1.5 cm was demonstrated [5]; this too should be interpreted with caution as water equivalent gel was used between bolus and phantom only for the 3D printed bolus, a significant advantage to introduce to the 3D printed bolus group. There is variation in the use of such gel in a clinical setting, and the impact on reproducible treatment set up, set up time, and patient comfort are unknown. Silicon hydrogel, a non‐rigid adhesive bolus, also demonstrated favourable conformity with a total air gap volume of 2.9cc [8]. The method of acquisition of data for 3D printed bolus also does not appear to significantly impact the presence of air gaps; surface scanner compared to a CT calculated bolus demonstrated minimal variation in air gaps at six anatomical sites including breast [7].

Clinically PLA bolus performed well compared to vinyl gel sheet, mean air gap with conventional bolus was 0.5 ± 0.3 cm, and 0.3 ± 0.3 cm for PLA in a study of 16 patients who were fitted for both types of bolus [16]. One outlier for the PLA group of 2.2 cm was reported, this was due to gradual but significant change in patient contour over the duration of radiation therapy. It should be noted that two patients were excluded from this study due to anatomical change between simulation and treatment delivery and one patient was excluded due to excessive respiratory motion causing motion artefact in the acquired cone beam CT. This highlights the role of appropriate patient selection when utilising a rigid bolus. Overall, anatomical conformity is difficult to compare across studies given the variation in measurement used.

Selection of bolus material may be relevant and further study in comparison of bolus material, such as thermoplastic polyurethane (TPU) which is less rigid in comparison to PLA, may be of benefit [24].

### 4.4 Dosimetry

Pre‐clinical photon studies are suggestive of increased surface dose with the use of 3D printed bolus compared to conventional bolus, if only by a small margin, across bolus material types. Studies demonstrated an increased surface dose of 4.2 cGy with PLA compared to conventional bolus [5] and an improved percentage dose difference between calculated and measured surface dose for PLA in 3‐ and 5‐mm thick bolus [10]. Silicone hydrogel also showed a small absolute dose difference, < 0.12%, and improved homogeneity and conformity indexes on assessment of the ratio between the volume covered by the reference isodose and the PTV.

Clinically, though the studies are limited by small patient numbers, they do demonstrate higher conformity and homogeneity indexes across different types of 3D printed bolus material. The variations between dose delivered and conformity index between PLA and conventional bolus were small in magnitude and higher for PLA [14, 16] and statistically significant [13]. TPE also demonstrated conformity and homogeneity, though this comparison was performed with historical data. Silica and epoxy injected mould 3D printed bolus also demonstrated slightly higher homogeneity and conformity indexes compared to conventional bolus [17]. The interpretation and comparison of these results across these studies is complicated by the small sample size and variable study design; a meaningful comparison between 3D printed bolus materials is not possible. However, overall, these findings are favourable for 3D printed bolus. There is minimal difference in surface dosimetry compared to TPS calculated surface dose demonstrated for conventional and 3D printed bolus, and excellent homogeneity and conformity.

Dosimetry with electron therapy was not well assessed, with only a small number of studies of unusual study design available. One study retrospectively calculated the tangential photon radiation therapy plan with modulated electron boost with and without 3D printed bolus and compared this to the previously delivered conventional photon radiation therapy [6]. This study design did not have a conventional bolus arm for comparison, instead comparing to no bolus and introducing another variable with a different treatment technique. As such, the improved conformity index demonstrated with 3D printed bolus is difficult to interpret. A small clinical study did demonstrate marginally higher mean conformity and homogeneity index with 3D printed PLA bolus in the context of electron therapy; however, it was limited by the small sample size of six patients [12].

Intra‐operative radiation therapy is an emerging option for breast radiation therapy with the aim to provide targeted therapy in lieu of whole breast radiation therapy and thus reduce toxicity of treatment. Early data are suggestive of non‐inferiority of ipsilateral breast recurrence in partial breast intra‐operative kilovoltage radiation therapy [25], though intraoperative electron radiation therapy showed slightly higher ipsilateral recurrence but no significant difference in overall survival [26]. Though this technique is yet to become routine clinical practice, the potential for 3D printed bolus in electron intra‐operative radiation therapy has been investigated. Moghaddam et al. [9] performed pre‐clinical investigations to review percentage depth dose (PDD) curves at different electron energies for PLA and water phantom with Monte Carlo calculation. The percentage increase in PDD with PLA in comparison to the water phantom alone was 0.9%, 0.4%, 0.8% and 0.2% for 6, 8, 10 and 12 MeV electron energies respectively. This early data is indicative that should intra‐operative electron radiation therapy be utilised in clinical practice, PLA may be a suitable bolus material to use, though further investigation will be required prior to use.

### 4.5 Dose Reduction to Organs at Risk

Reduction of OAR dose is an important aspect of improving treatment toxicity. In the pre‐clinical setting, dose to OARs were considered within the anthropomorphic phantom or retrospectively calculated for previously treated patients. All pre‐clinical trials met recommended local departmental constraints which were in keeping with RTOG OAR constraints [27]. There was a demonstrated a reduction in left anterior descending (LAD) artery *D*_max_ by almost 3% and reduction in ipsilateral lung V_5_ and V_20_ by less than 4% and 2% respectively when comparing a 3D bolus modulated electron boost to a conventional electron boost in a single study. However, the conventional electron boost arm did not utilise any bolus as per the local treatment protocol. This was due to small breast size in the Chinese population included in the study. As such, the reduction to OAR dose demonstrated in this trial cannot be specifically attributed to 3D printed bolus. A separate study comparison PLA and conventional bolus did demonstrate a marginal reduction in ipsilateral lung doses [5]. A comparison of silicone Hydrogel bolus to virtual CT bolus and demonstrated a < 2.4% dosimetric difference to OAR [8], though this is difficult to interpret in the absence of a standard bolus comparator.

In the clinical setting, Yang et al. [15] compared electron therapy with 3D printed bolus to conventional photon therapy in 28 patients and demonstrated a reduction in mean and maximum heart doses and mean, V5 and V20 ipsilateral lung doses. This study design introduced a treatment technique variable, reduced dose at depth is expected with the use of electrons compared to megavoltage photons and thus the relevance or impact of 3D printed bolus in this case is unclear. Zhang et al. [13] showed an on average marginal but statistically significant reduction of the mean heart dose (*p* = 0.035) and the ipsilateral lung dose (*p* < 0.001) when comparing PLA compared to standard vinyl gel sheet. A small six patient study compared conventional to 3D printed PLA bolus and demonstrated a reduction in mean and median lung doses in the 3D printed bolus arm [12]. Overall, it is difficult to draw conclusions on OAR reduction with the use of 3D bolus given small sample size and study design introducing other variables which impact causality of results.

### 4.6 Toxicities

Changes to dose delivery to skin and superficial tissues may result in increased toxicities including radiation dermatitis and pneumonitis. Radiation dermatitis is a commonly experienced toxicity for radiation therapy in this setting [28]. Four clinical studies investigated the toxicities related to radiation treatment in the post‐mastectomy radiation therapy (chest wall) setting. PLA demonstrated a small but statistically insignificant lower rate of radiation dermatitis [13]; rates of toxicities reported varied from a single patient with radiation dermatitis to 41.67% of assessed patients presenting with Grades 2–4 radiation dermatitis. It is difficult to draw conclusions based on the results of this data as only one trial, Zhang et al. [13], had a standard of care traditional bolus comparator arm. However, this study did include 75 patients, and outcome data is in keeping with radiation dermatitis toxicities expected with post‐mastectomy radiation therapy [22]. A study of 3D printed TPE bolus showed four of 30 patients experienced radiation dermatitis; again, patient numbers are small, and comparison was made to historical conventional data in this study [11]. Interestingly, the electron density of the TPE bolus was reported at 0.83, and the PLA bolus was 1.11. Variation in electron density may impact surface dose and subsequent toxicities.

Radiation pneumonitis is a rare complication of radiation therapy in the post‐mastectomy setting and was only assessed by Zhang et al. [13] who reported Grade 1 toxicity in 20% compared to 14.3%, and Grade 2 toxicity in three compared to zero patients for the conventional and 3D printed bolus groups respectively. These rates of radiation pneumonitis are notably higher than what would be expected in clinical practice (2.4%) [29], though not statistically significant (*p* = 0.466). The percentage of patients found to have pneumonitis may reflect increased surveillance for complications as the patients were followed up 12 weeks post completion of radiation therapy to assess for pneumonitis, it was not specified whether this review included a CT scan.

### 4.7 Limitations

Despite the use of PRISMA, it is possible that there are articles which were not indexed in the databases used or by the pre‐determined search terms utilised. This is particularly pertinent in the setting of an intervention which is relevant to many members of the multidisciplinary team, including physicists, radiation therapists and radiation oncologists. Multidisciplinary reviewers were included to minimise this risk. The potential for publication bias is also acknowledged, with favourable publishing of data demonstrating efficacy of 3D printed bolus. We recognise the majority of studies included investigated the use of 3D printed bolus in Asian cohorts, and there may be a difference in patient size and treatment to consider when applying these findings to varied populations and treatment protocols. Notably, patient or breast size has only been quantified in one of the studies included. It is difficult to draw firm conclusions from the data that is available at this time, given the limited sample size, variable study design and variable comparator arm.

## 5 Conclusion

This scoping review has comprehensively reviewed the available literature for 3D printed bolus in the setting of breast radiation therapy. Overall, many studies report favourable results in dosimetry, anatomic conformity, OAR dose distribution and toxicities. These findings are suggestive that 3D printed bolus is similar to conventional bolus in performance and has reasonable application in workflow and cost. However, these results should be interpreted with caution as there is limited data comparing 3D printed bolus to conventional bolus in the clinical setting, and where available this research is limited by small sample size and variable study design. Furthermore, the results available were largely reviewing PLA 3D printed bolus, not accounting for the variety of 3D printed bolus materials available such as TPU. This review may guide further research in 3D printed bolus for breast radiation therapy to facilitate applicability of its use in clinical practice.

## Conflicts of Interest

The authors declare no conflicts of interest.

## Data Availability

Research data are not shared.
